# Comparative analysis of prophages carried by human and animal-associated *Staphylococcus aureus* strains spreading across the European regions

**DOI:** 10.1038/s41598-021-98432-8

**Published:** 2021-09-23

**Authors:** Romen Singh Naorem, Gunajit Goswami, Schneider Gyorgy, Csaba Fekete

**Affiliations:** 1 Department of General and Environmental Microbiology, Institute of Biology and Sport Biology, University of Pécs, Ifjusag utja. 6, Pecs, 7624 Hungary; 2 Multidisciplinary Research Unit, Jorhat Medical College and Hospital, Jorhat, Assam India; 3 Department of Medical Microbiology and Immunology, Medical School, University of Pécs, Pecs, Hungary

**Keywords:** Phylogeny, Bacteria, Bacteriophages, Microbial genetics, Comparative genomics, Mobile elements

## Abstract

*Staphylococcus aureus* is a major human and animal pathogen although the animal-associated *S. aureus* can be a potential risk of human zoonoses. Acquisition of phage-related genomic islands determines the *S. aureus* species diversity. This study characterized and compared the genome architecture, distribution nature, and evolutionary relationship of 65 complete prophages carried by human and animal-associated *S. aureus* strains spreading across the European regions. The analyzed prophage genomes showed mosaic architecture with extensive variation in genome size. The phylogenetic analyses generated seven clades in which prophages of the animal-associated *S. aureus* scattered in all the clades. The *S. aureus* strains with the same *SCCmec* type, and clonal complex favored the harboring of similar prophage sequences and suggested that the frequency of phage-mediated horizontal gene transfer is higher between them. The presence of various virulence factors in prophages of animal-associated *S. aureus* suggested that these prophages could have more pathogenic potential than prophages of human-associated *S. aureus.* This study showed that the *S. aureus* phages are dispersed among the several *S. aureus* serotypes and around the European regions. Further, understanding the phage functional genomics is necessary for the phage-host interactions and could be used for tracing the *S. aureus* strains transmission.

## Introduction

*Staphylococcus aureus* is a major human and animal pathogen, leading to cause severe hospital-acquired, community-acquired, and animal-acquired infections^1^. *S. aureus* colonies in various ecological niches within human and animal hosts^2^ and causing the diverse ranges of infections ranging from skin and soft tissue infections to life-threatening infections^3,4^. The animal-associated *S. aureus* can be a potential risk of human zoonoses and a threat to human public health^5,6^. The genomic plasticity of *S. aureus* facilitates the acquisition of the mobile genetic elements (MGEs) such as prophage, *S. aureus* pathogenic island (SaPIs), genomic islands (νSa), transposons, staphylococcal cassette chromosomes (SCCs), and plasmids, which have an array of genes encoding proteins involved in antibiotic resistance, virulence, and other contingency functions^7–9^. Acquisition of prophages or phage-related genomic islands determine the diversity of the *S. aureus* species and contribute to a dramatic form of genetic adaptation to various host conditions^10^. Based on the genome sequence and sizes of the *S. aureus* phages, phages can be grouped into three classes among *Caudovirales* order, viz*., Podoviridae* family belongs to class I with the smallest genome (< 20 kb), *Siphoviridae* family belongs to class II showing intermediate genome sizes (39–125 kb), and *Myoviridae* family belongs to class III with largest genome size (> 125 kb)^2,11^. The genome of *Siphoviridae* family is composed of six functional modules viz., lysogeny, DNA replication, packaging, head, tail, and lysis^12^. Prophage can be switched from a lysogenic state to a lytic state in response to the metabolic state or environmental stresses of the host^13^. The expression of a specific phage repressor gene (*cI*) inhibits the transcription of the genes required for the lytic cycle and the prophage becomes quiescent. The CI repressor also inhibits the integration of other phage genomes of the same group and confers immunity to superinfection^14^. Phages or prophage-like elements contribute to horizontal transfer of pathogenicity islands which carry virulence factor-encoding genes (VFGs) such Panton-Valentine leukocidin (PVL encodes *lukFS-PV* genes), the immune evasion cluster (IEC) associated with human specificity (*chp, sak,* and *scn*), exfoliative toxins (*eta and etb*) and enterotoxins (*sea, see, seg, sek,* and *sep*)^15–20^. It is reported that phages 80α and 80 can mobilize a variety of superantigen-encoding pathogenicity islands SaPI1 and SaPI2, respectively^21,22^, and *pvl* genes are encoded by prophages phiPVL and phiSLT^23^. The mobilization of phages among the *S. aureus* strains has increased the frequency of intra-strain and inter-strain exchange of VFGs or resistance genes that helps to adapt in diverse hostile environments and contributes to pathogenesis and evolution^20,21,24^.

In this study, we performed an *in-silico* screening and identification of prophages in the genomes of *S. aureus* strains which were reported to be associated with human and animal infections in different European regions. We compared the variation in genome architecture and genetic diversity among the identified prophage genomes. In addition, we studied the nature of distribution and the relationship of prophages harbored by human and animal-associated *S. aureus* strains concerning *SCCmec*, clonal complex, host infected sites, and geographic locations.

## Results

To study prophages diversity, genome sequences of *S. aureus* strains associated with human (*Homo sapiens*) infections (n = 34), bovine (*Bos tourus*) infections (n = 22), and dog (*Canis lupus familiaris*) infections (n = 4) were retrieved from NCBI database. The geographical locations of the selected *S. aureus* strains were shown in Figure S1. The list of genomes and their respective features are shown in Table S1.

### Identification and general genomic features of S. aureus prophages

PHASTER identified a total of 170 prophages of which 101 prophages (46 complete, 14 questionable, and 41 incomplete) were extracted from the genomes of *S. aureus* associated with human infections, 59 prophages (16 complete, 8 questionable, and 35 incomplete) were extracted from the genomes of *S. aureus* associated with *B. tourus* infections, and 10 prophages (3 complete, 6 questionable, and 1 incomplete) were extracted from genomes of *S. aureus* associated with *C. lupus familiaris* infections. The distribution of prophages in the *S. aureus* genome is represented in Figure S2. The 65 intact/complete prophages were selected based on PHASTER scores (Table S2). All the identified complete prophages were belonged to *Siphoviridae* family and having temperate lifestyles. Among the 65 analyzed prophages, 57 prophages were extracted from methicillin-resistance *S. aureus* (MRSA) strains, while the 8 analyzed prophages were extracted from methicillin-sensitive *S. aureus* (MSSA) strains in which 4 prophages were from 4 MSSA strains (*S. aureus* HD1410, *S. aureus* I3, *S. aureus* SA13-192, and *S. aureus* SA14-639)-associated with human infections, and another 4 prophages were extracted from 4 MSSA strains (*S. aureus* 483, *S. aureus* 909, *S. aureus* C3489, and *S. aureus* C5086)-associated with bovine and dog infections. Among the MRSA strains, SA G6, and *S.* SA G8 strains were reported earlier as hospital-associated MRSA (HA-MRSA)^25^, while the other MRSA strains carried Staphylococcal Cassette Chromosome *mec* (*SCCmec*) types IV, and V which are regarded as community-associated-MRSA (CA-MRSA)^26^. The MLST analysis result revealed that *S. aureus* strains associated with the animal infections have sequence types ST398 (clonal complex, CC398) and ST151 (CC151), while the *S. aureus* strains associated with human infections have ST398 (CC398), ST8 (CC8), ST5 (CC5), etc.(Table S2). The genome sizes of intact/complete prophages were in the range of approximately 24.8–87.8 kb, and the GC content varied between 32.16 and 35.38%. The highest number (132) of coding sequence (CDS) was found in phiG4-3 (*S. aureus* SA G6) and the lowest number (29) of CDS was found in phiH7-2 (*S. aureus* strain H7).

### Sequence clustering and phylogenetic relationship of the prophages

**Figure 1:**
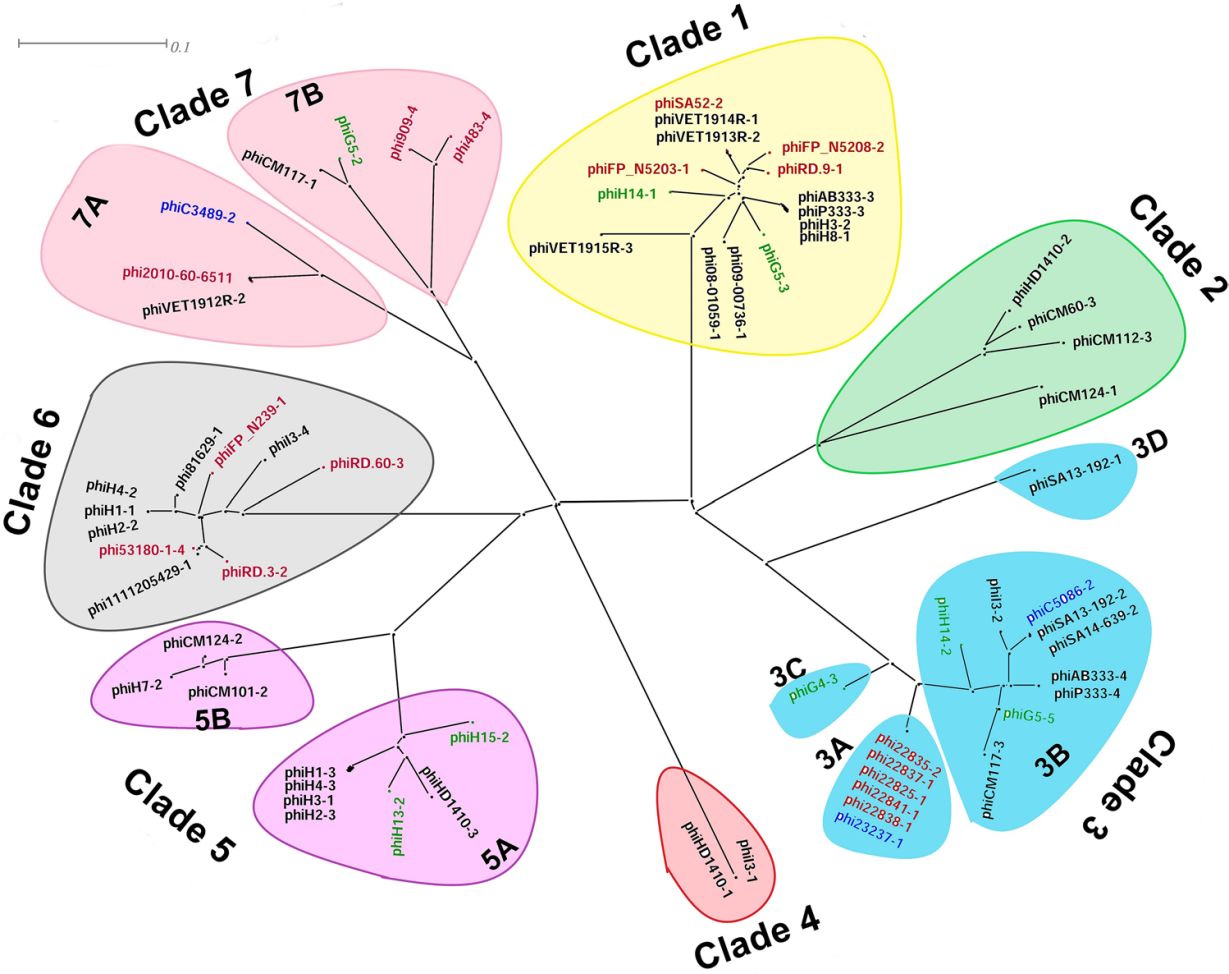
Cluster generated from the prophage genome sequences and phylogenetic relationship. The clades were shaded with different colors and the prophage names labeled in black, red, and blue colors represent the prophages extracted from the *S. aureus* associated with *Homo sapiens*, *Bos tourus*, and *Canis lupus familiaris*, respectively. And the prophage names labeled in green color represent the prophages extracted from our previous study^25,27^.

Sequence clustering was performed by aligning the whole-genome sequences of 65 intact/ complete prophage genomes carried by human and animal-associated *S. aureus*. The generated phylogenetic tree grouped the prophages into 7 different clades (Fig. 1; Table 1). The prophages carried by human and animal-associated *S. aureus* strains linked with different infected sites and different geographical locations were dispersed randomly in all clades. Although, it was expected that the prophages of animal-associated *S. aureus* and human-associated *S. aureus* strains would from individual clusters, this was not the case. There was no correlation between the clade, the host, site of infection, or geographical location.

**Table 1:** Distribution of prophages of *S. aureus* into different clades obtained from the phylogenetic analysis.

Sl. nos	Clade	Sub clade	Phage genomes	Common CDS features
1	Clade 1	–	phiG5-3, phi09-00736-1, phi08-01059-1, phiH14-1, phiH8-1, phiAB333-3, phiP333-3, phiH3-2, phiSA52-2, phiVET1913R-2, phiVET1914R-1, phiFP_N5208-2, phiFP_N5203-1, phiRD.9-1, and phiVET1915R-3	Phage hypothetical proteins (HP), phage proteins (PP), tail protein (TP), tail tape measure protein (TTmP), major tail protein (mTP), major tail protein, head–tail joining protein (HTJP), DNA packaging protein (DNA_PP), capsid protein (CapP), caseinolytic protease (Clp), portal protein (portP), terminase large subunit (TerL), terminase small subunit (TerS), HNH endonuclease, transcriptional activator rinB (TransA), virulence-associated E family protein (VirE), and DNA-binding protein
2	Clade 2	–	phiHD1410-2, phiCM60-3, phiCM112-3, and phiCM124-1	Hypothetical proteins (HP), phage protein (PP), holin, tail fiber protein, N-acetylglucosaminidase (N-AGA), minor structural protein (mSP), and transcriptional activator rinB (TransA)
3	Clade 3	3A	phi22825-1, phi22838-1, phi22835-2, phi22841-1, phi22837-1, and phi23237-1	Hypothetical proteins (HP), phage proteins (PP), tail protein (TP), tail tape measure protein (TTmP), major tail protein (mTP), head–tail joining protein (HTJP), DNA packaging protein (DNA_PP), terminase small subunit (TerS), capsid protein (CapP), Clp protease, portal protein (portP), terminase large subunit (TerL), HNH endonuclease, transcriptional activator (TransA), PVL, antirepressor protein (AntiR), integrase, phospholipase C (Hlb), leukocidin/hemolysin toxin family protein (LukG/Hlg), minor structural proteins (mSP), holin, amidase, staphylokinase (SAK), SH3 domain-containing protein, Transposase binding protein, dUTPases, staphylococcal complement inhibitor (SCIN), chemotaxis inhibitory protein (CHIPS), transcriptional regulator (TransR), PemK like phage protein, and leukocidin/lemolysin toxin family protein (LukH)
		3B	phiI3-2, phiSA14-639-2, phiSA13-192-2, phiC5086-2, phiAB333-4, phiP333-4, phiG5-5, phiCM117-3, and phiH14-2	Hypothetical proteins (HP), phage proteins (PP), SCIN, SH domain-containing protein, chemotaxis inhibitory protein (CHIPs), amidase, holin, minor structural protein (mSP), tail protein (TP), tail tape measure protein (TTmP), major tail protein (mTP), head–tail joining protein (HTJP), DNA packaging protein (DNA_PP), terminase small subunit (TerS), capsid protein, Clp protease, portal protein, terminase large subunit (TerL), HNH endonuclease, transcriptional activator (TransA), dUTPase, PVL, transposase-associated ATP/GTP binding protein, and transcriptional regulator
		3C	phiG4-3	–
		3D	phiSA13-192-1	–
4	Clade 4	–	phiI3-1, and phiHD1410-1	Hypothetical proteins (HP), cyclase enzyme, metE, metH, bifunctional cystathionine gamma-lyase/gamma-synthase, parB, MscS family small conductance mechanosensitive ion channel protein, YchF, RpsF, RpsR, single-strand DNA binding protein (SSBP), PemK-like growth inhibitor, pathogenicity island protein (integrase), DNA-binding protein, DNA-binding protein, pathogenicity island DNA-binding protein, bovine pathogenicity island protein, mobile element-associated protein, primase, pathogenicity island protein, phage protein, mobile element-associated protein, spore coat protein, terminase small subunit (TerS), abortive infection bacteriophage resistance protein, integrase, YxeA family protein, and secreted protease inhibitor
5	Clade 5	5A	phiH15-2, phiHD1410-3, phiH13-3, phiH1-3, phiH3-1, phiH4-3, and phiH2-3	Hypothetical proteins (HP), phage proteins (PP), transcriptional activator (TransA), HNH endonuclease, terminase large subunit (TerL), portal protein (portP), Clp protease, capsid protein (CapP), terminase small subunit (TerS), DNA packaging protein (DNA-PP), head–tail joining protein (HTJP), major tail protein (mTP), tail tape measure protein (TTmP), tail protein (TP), minor structural protein (mSP), amidase, staphylokinase (SAK), and SH3 domain-containing protein
		5B	phiCM101-2, phi124-2, and phiH7-2	Phage proteins (PP), hypothetical proteins (HP), HNH endonuclease, terminase large subunit (TerL), head–tail joining protein (HTJP), tail tape measure protein (TTmP), tail protein (TP), minor structural proteins (mSP), holin, amidase, and staphylokinase (SAK)
6	Clade 6	–	phi53180-1-4, phi1111205429-1, phiRD.3-2, phiFP_N239-1, phi81629-1, phiH4-2, phiH1-1, phiH2-2, phiI3-4, and phiRD.60-3	Hypothetical proteins (HP), phage protein (PP), transcriptional activator RinB (TransA), virulence-associated E family protein (virE), VRR-NUC domain protein, HNH endonuclease, terminase small subunit (TerS), terminase large subunit (TerL), portal protein (portP), Clp protease, major capsid protein (CapP), DNA packaging protein (DNA_PP), head–tail joining protein (HTJP), major tail protein (mTP), tail protein (TP), and tail tape measure protein (TTmP)
7	Clade 7	7A	phiC3489-2, phiVET1912R-2, and phi2010-60-6511	Hypothetical proteins (HP), phage proteins (PP), transcriptional activator (TransA), terminase small subunit (TerS), terminase large subunit (TerL), Portal protein (portP), SPP1 family, Minor head protein (MHP), Head–tail adaptor (HTA), Tape measure proteins (TmP), Tail protein (TP), minor structural protein (mSP), N-acetylglucosaminidase (N-AGA), and tail fiber protein (TFP)
		7B	phiG5-2, phiCM117-1, phi909-4, and phi483-4	Hypothetical proteins (HP), phage proteins (PP), ssDNA-binding protein, PVL, dUTPase, transcriptional activator (TransA), terminase small subunit (TerS), Portal protein (portP), SPP1 family, head morphogenesis protein, terminase large subunit (TerL), tape measure protein (TmP), tail protein (TP), minor structural protein (mSP), N-acetylglucosaminidase (N-AGA), and tail fiber protein (TFP)

### Comparative genome analyses of S. aureus prophages

**Figure 2:**
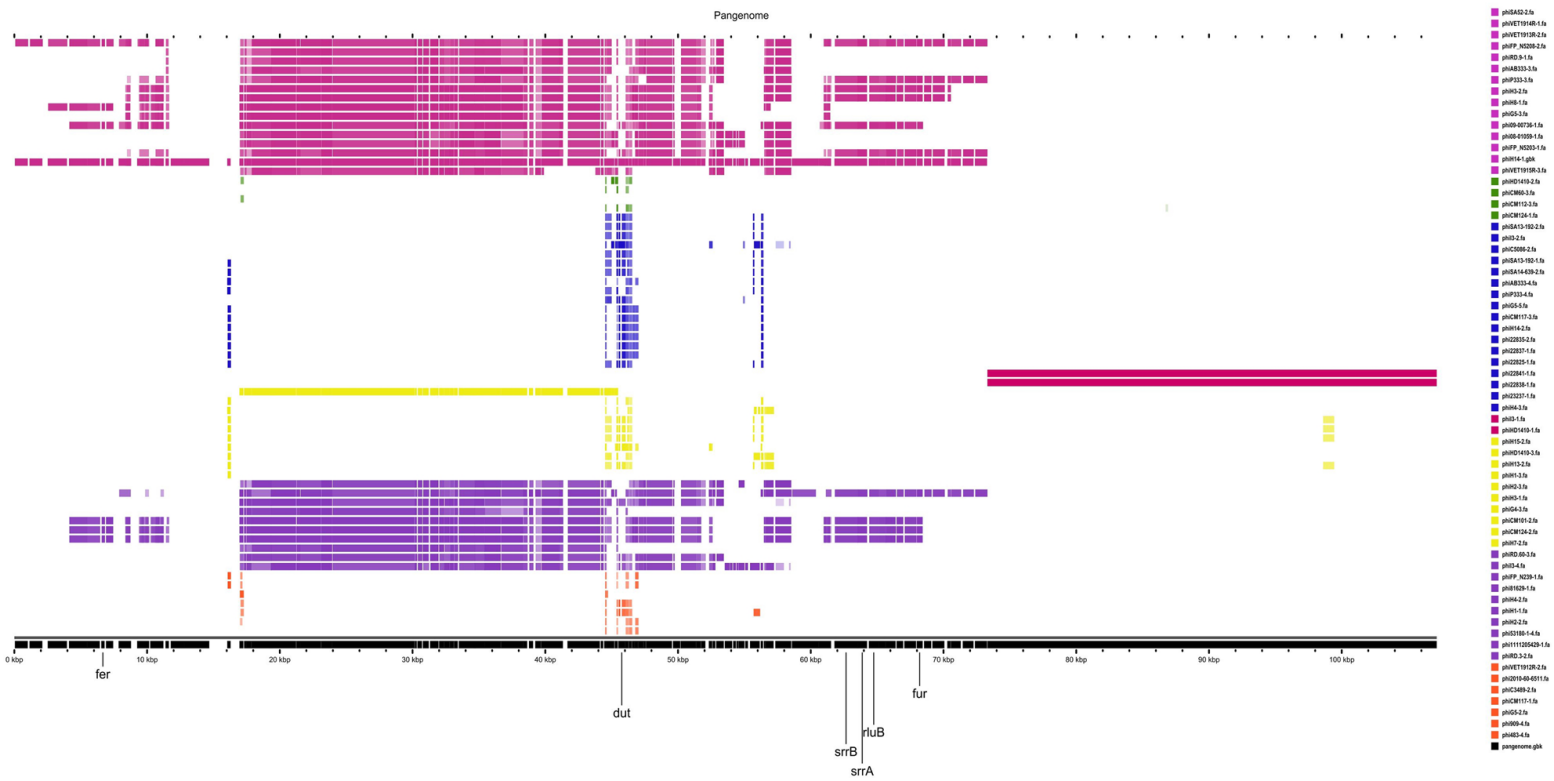
Pan-genome analysis of genome of the prophages. The pan-genome was generated as linear map with brown color below the background line (black), each linear genome sequence represents an individual putative prophage and are color-coded clade wise (clade 1 = marron, clade 2 = green, clade 3 = blue, clade 4 = dark red, clade 5 = yellow, clade 6 = purple, and clade 7 = orange).

The pan-genome analysis of 65 intact prophages of *S. aureus* is represented in Fig. 2. The reference pan-genome was found to be 107,158 bp in length with 90 CDS features, however, a core-genome was not observed, and the accessory genomes of all individual prophages were unique. This signified that all the prophages were variants in their genetic components. Clade/subclade-wise pan-genome analysis was executed to find the shared genes within the clade/subclade and the observations are given below:

### Staphylococcus aureus prophages of clade 1

The clade 1 encompasses 15 prophages with a total sequence size of 122,268 bp encoding 168 CDS features. The sequences shared 19.84% similarities, and the similar region encodes 28 common CDS features (Fig. 1; Table 1).

**Figure 3:**
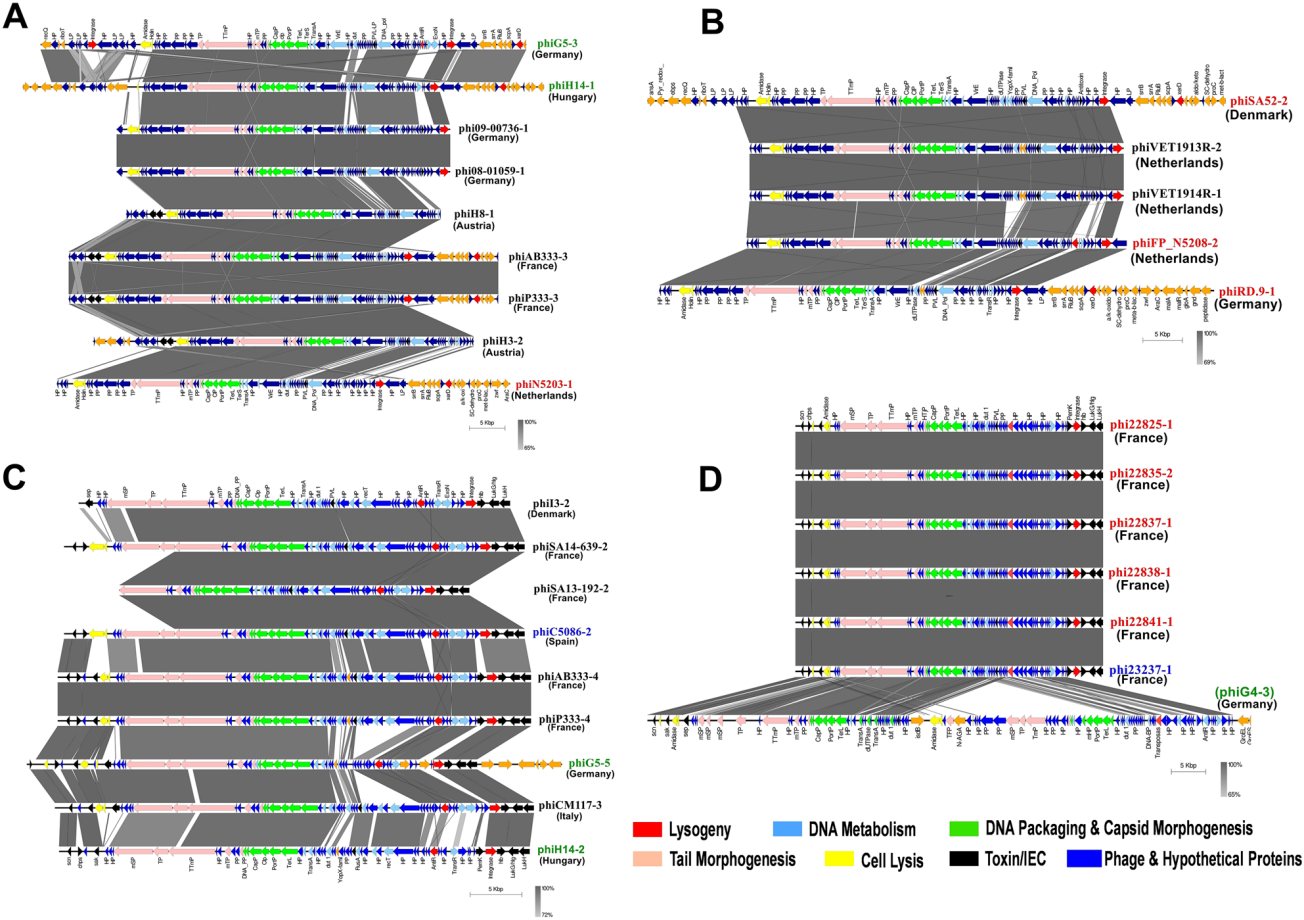
The mosaic architecture of *S. aureus* prophage genomes. (**A**) Comparison of prophages of clade 1, representing phiG5-3, and phiH14-1 sequenced of our previous study^25^ and 7 prophages associated with *S. aureus* reported from Germany, Austria, France, and Netherlands; (**B**) Comparison of prophages of clade 1, representing prophages of *S. aureus* associated with *Homo sapiens* and *Bos tourus*; (**C**) Comparison of prophages of clade 3, representing prophages phiG5-5 & phiH14-2 (subclade 3B) sequenced of our previous study and 6 prophages associated with *S. aureus* reported from Denmark, France, Spain, Germany, Italy, and Hungary; (**D**) Comparison of prophages in clade 3, representing prophages phiG4-3 (subclade 3C) sequenced of our previous study and 6 prophages (subclade 3A) associated with *S. aureus* spreading in France, Spain, and Germany. Phages and country of origins are indicated on the right (Prophage names labeled in black, red, and blue color indicated prophages associated with *Homo sapiens*, *Bos tourus,* and *Canis lupus familiaris* respectively. And prophage names labeled in green color indicated the prophages extracted from our previous study^25,26^) and grey shaded regions are homology regions.

In this clade, phiSA52-2 showed distinctive CDS features and possesses the highest number of accessory genes (67 CDS) while, phiH8-1 has the lowest number of accessory genes (30 CDS) (Fig. 3A,B; clade 1 of Figure S3).

### Staphylococcus aureus prophages of clade 2

With a total sequence size of 112,518 bp that encodes 149 CDS features, clade 2 has 4 prophage sequences. The sequences shared 6.11% similarity that encodes 7 common CDS features (Fig. 1; Table 1). In this clade, phiCM124-1 showed distinctive features compared with the other prophage sequences and possessed the highest number of accessory genes (80 CDS). The lowest number of accessory genes (28 CDS) was observed in phiCM112-3.

### Staphylococcus aureus prophages of clade 3

The subclade 3A comprised of 6 prophage sequences and the phage sequences shared 100% sequence similarity which encodes 69 CDS features. Among the 6 prophages, phi23237-1 was from a different host spectrum, since its host is associated with dog infection while the hosts of the other 5 prophages are associated with bovine infections (Figs. 1, 3D; Table 1). The subclade 3B contains 9 prophage sequences with a total sequence size of 74,025 bp that encode 111 CDS features. It showed 27.23% bp similarity that encodes 29 common CDS features (Figs. 1, 3C; Table 1). The maximum number of accessory genes (40 CDS) was observed in the phiG5-5 while, phiSA13-192-2 occupied the lowest number of accessory genes (24 CDS) (clade 3B of Figure S3). The phiG4-3 and phiSA13-192-1 were distinct from other subclades 3A, and 3B resulting in discrete subclades 3C, and 3D, respectively (Fig. 1; Table 1).

### Staphylococcus aureus prophages of clade 4

The clade 4 contained 2 prophage sequences with a total sequence size of 33,850 bp. Although both prophages shared 100% sequence similarity, they have different host spectra, since their hosts belonging to different geographical regions, and infects at different body sites. The shared sequences have 45 CDS features (Fig. 1; Table 1).

### Staphylococcus aureus prophages of clade 5

**Figure 4:**
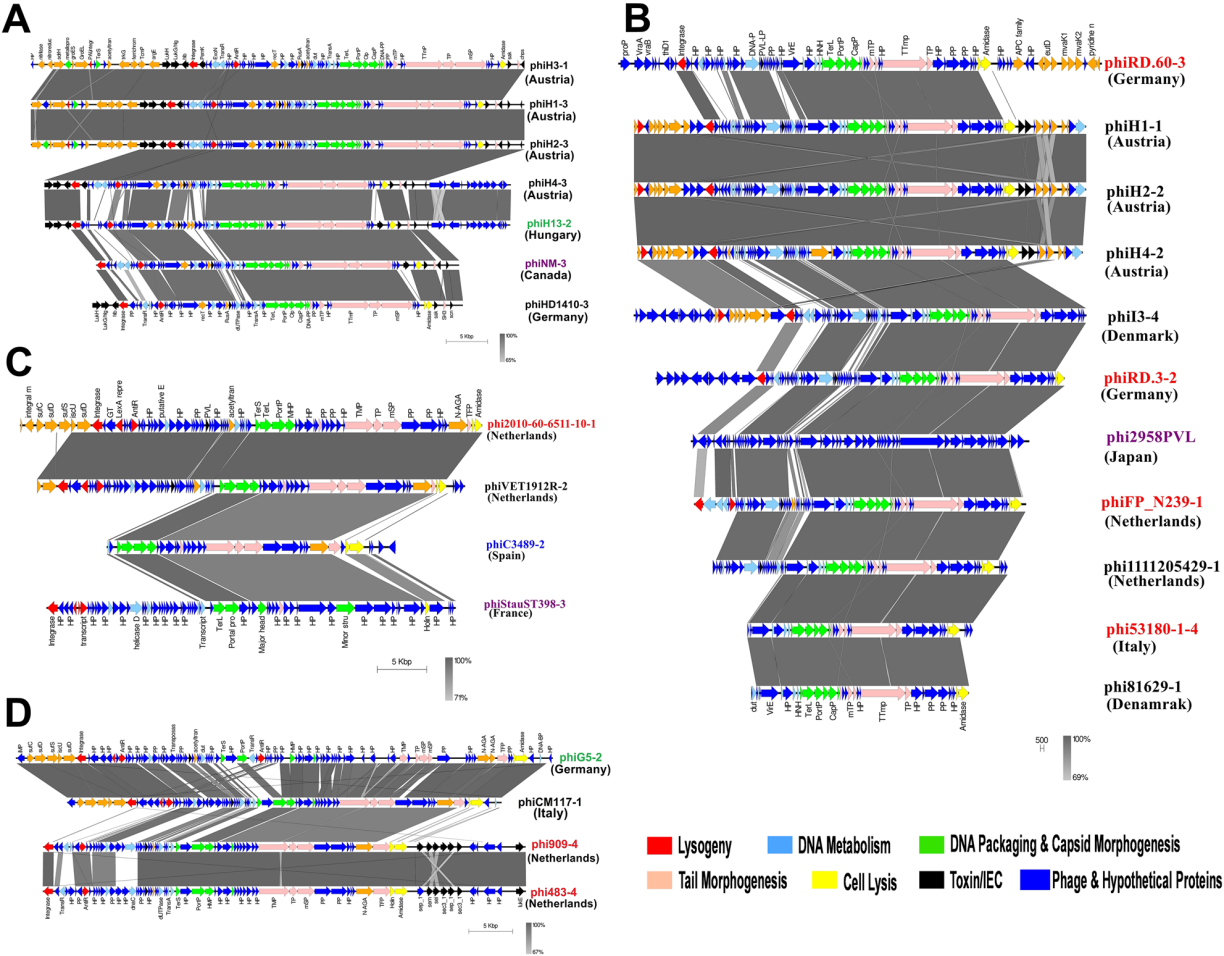
The mosaic architecture of *S. aureus* prophage genomes. (**A**) Comparison of prophages of clade 5, representing the prophages of subclade 5A, and phiH13-2 sequenced of our previous study^27^ reported from Austria, Hungary, Canada (reference prophage), and Germany; (**B**) Comparison of prophages of clade 6, representing prophages of *Homo sapiens* and *Bos tourus -*associated *S. aureus* spreading in Germany, Austria, Denmark, Netherlands, Japan (reference prophage), and Italy; (**C**) Comparison of prophages of clade 7, representing subclade 7A prophages *Homo sapiens*, *Bos tourus,* and *Canis lupus familiaris*-associated *S. aureus* reported from Netherland, Spain, and France; (**D**) Comparison of prophages of clade 7, representing phiG5-2 prophage sequence of our previous study^25^ and 3 prophages of subclade 7B associated with *S. aureus* reported from Italy, and Netherland. Phages and country of origins are indicated on the right (the prophage names labeled in black, red, blue, and purple color indicate prophages associated with *Homo sapiens*, *Bos tourus, Canis lupus familiaris,* and reference, respectively. And prophage names labeled in green color indicated the prophages extracted from our previous study^25,27^ and grey shaded regions are homologous regions.

The subclade 5A contained 7 prophage sequences with 119,097 bp total sequence size which encodes 159 CDS features (Fig. 1). The sequences in this subclade showed 17.64% sequence similarities and share 23 CDS features (Table 1). In this subclade, the highest accessory genes were found in the phiH3-1 (75 CDS), and lowest in phiHD1410-3 (59 CDS) (clade 5A of Figure S3). In this subclade, phiH1-3 and phiH2-3 showed the highest sequence identity, whereas phiHD1410-3 showed closed sequence similarity with the reference phiNM-3 (Fig. 4A). Subclade 5B consist of 3 prophage sequences that shared 30.36% sequence similarities out of a total sequence size of 77,303 bp and having 27 common CDS features (Fig. 1; Table 1). In this clade, the phiH7-2 sequence has the lowest number of accessory genes.

### Staphylococcus aureus prophages of clade 6

The clade 6 contained 10 prophage sequences with a total sequence size of 133,139 bp encoding 170 CDS features (Fig. 1; Table 1). It showed 20.81% sequence similarity that encodes 29 CDS features (Fig. 4B; clade 6 of Figure S3). The phiRD.60-3 has the highest number of accessory genes (57 CDS) while the phi81629-1 has the lowest number of accessory genes (5 CDS). The phiH4-2, phiH1-1, and phiH2-2 shared 99.77% sequence similarities with 93 common CDS features (Fig. 4B). The prophages, phi81629-1, and phi53180-1-4 were found closest to each other. Moreover, phiFP_N239-1 and phi2958PVL showed high sequence identity (Fig. 4B).

### Staphylococcus aureus prophages of clade 7

The subclade 7A contained 3 prophage sequences with 56,344 bp total sequences that share 38.03% sequence similarities which encode 25 CDS features out of 86 total CDS features (Fig. 1; Table 1). The phiC3489-2 displayed the lowest number of CDS features (10), while the phi2010-60–65,511-10-1 showed the highest number of CDS features (48) (Fig. 4C; clade 7A of Figure S3). The reference phiStauST398-3 showed high sequence identity with phiVET1912R-2 (Fig. 4C). Subclade 7B consists of 4 prophage sequences having 98,346 bp that codes for 115 CDS features. This subclade shared 26.35% similarities that encode 45 CDS features (Figs. 1, 4D; Table 1; clade 7B of Figure S3). Glycosyltransferase family protein was detected in phi2010-60-65511-10–1 and phVET1912R-2 prophage sequences. This protein performs the conversion of host serotype during lysogeny of temperate phage^28,29^.

### Putative virulence factors associated with S. aureus prophages

**Figure 5:**
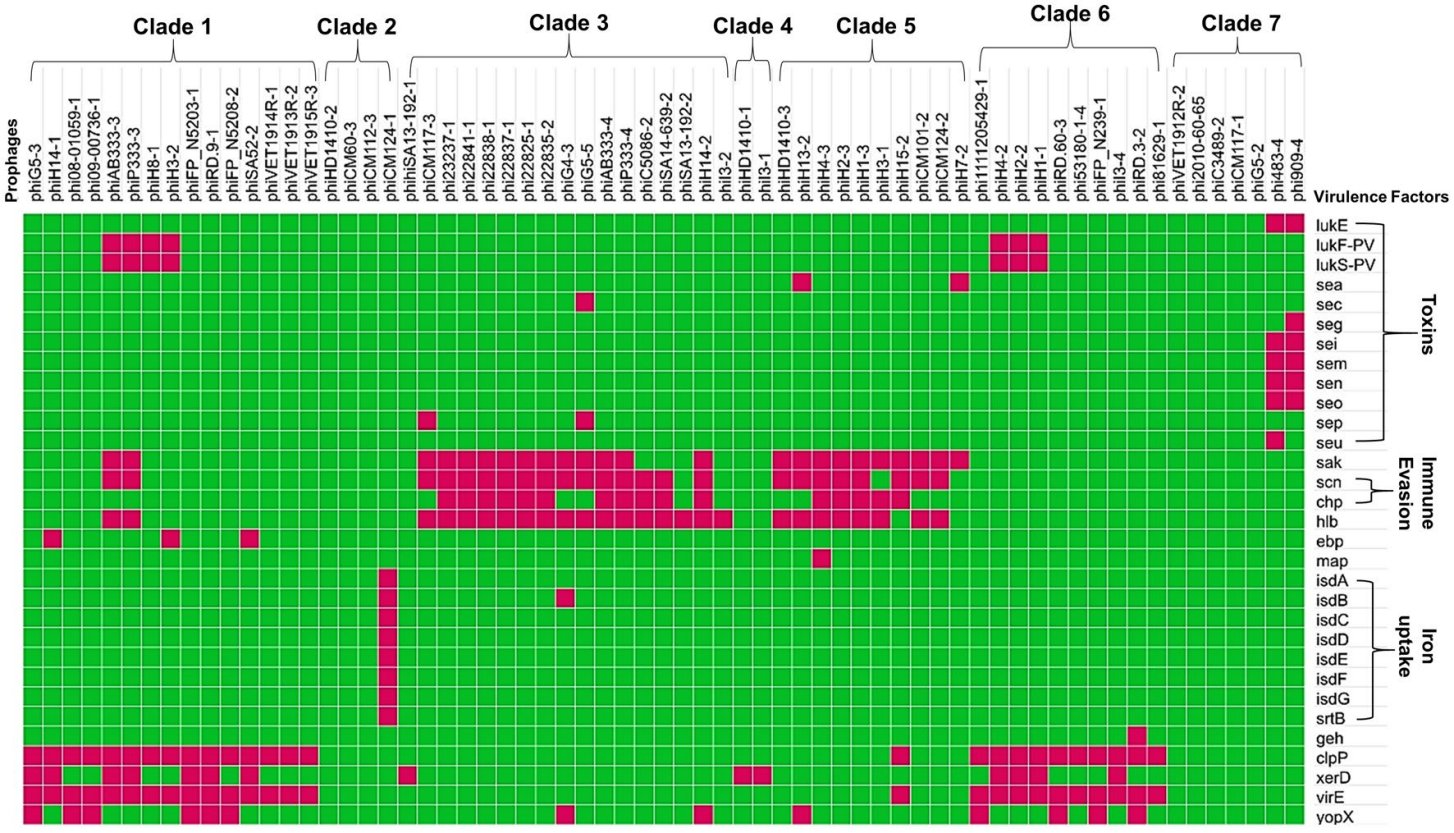
Heatmaps showing the presence/absence of virulence genes in the prophages. The top labels indicate the name of the prophages and their clades (bold). The right labels indicate the name of the virulence gene and their categories (bold). The presence and absence of virulence genes are indicated by red and green colors, respectively*.*

The prophages of *S. aureus* associated with human and animal infections were found to harbor virulence factor encoding genes that may play a role in immune evasion, tissue evasion, toxins, adherence, and iron uptake or may code for toxins. The comparative analyses of virulence factor encoding genes associated with prophages are summarized in a heatmap (Fig. 5; Table 2). Clades 3 and 5 showed the highest occurrence of immune evasion genes. The highest prevalence of toxin encoding genes was observed in clade 7. The lowest prevalence of virulence encoding genes was observed in clades 2 and 4.

**Table 2:** Details of the putative virulence factors associated with prophages of *S. aureus.*

Clade	Prophage	Putative virulence factor
1	All prophages	*clpP,* and *virE* genes
	phiH14-1, phiSA52-2, phi08-01059-1, phi09-00736-1, phiFP_N5208-2, phiG5-3, phiRD.9-1, phiFP_N5203-1, phiVET1914R-1, phiVET1913R-2, and phiVET1915R-3	*lukF-PV,* and *lukS-PV* genes
	phiH8-1, phiH3-2, phiH14-1, phiSA52-2, phi08-01059-1, phi09-00736-1, phiFP_N5208-2, phiG5-3, phiRD.9-1, phiFP_N5203-1, phiVET1914R-1, phiVET1913R-2, and phiVET1915R-3	*sak, scn,* and *hlb* genes
	phiAB333-3, phiP333-3, phiH8-1, phi08-01059-1, phi09-00736-1, phiFP_N5208-2, phiG5-3, phiRD.9-1, phiFP_N5203-1, phiVET1914R-1, phiVET1913R-2, and phiVET1915R-3	*eap* gene
	phiH8-1, phiH3-2, phi08-01059-1, phi09-00736-1, phiFP_N5208-2, phiVET1914R-1, phiVET1913R-2, and phiVET1915R-3	*xerD* gene
	phiAB333-3, phiP333-3, phiH8-1, phiH3-2, phiH14-1, phiSA52-2, phiVET1914R-1, phiVET1913R-2, and phiVET1915R-3	*yopX* gene belonging to Type-III secretion system
2	phiCM124-1	iron-regulated surface determinants (*isd*) gene clusters (*isdA-isdH)*
3	All prophages	*hlb* gene
	phiAB333-4, phiP333-4, phiCM117-3, phiG5-5, phiH14-2, phi23237-1, phi22825-1, phi22837-1, phi22838-1, phi22835-2, phi22841-1, and phiG4-3	*sak* gene
	phiAB333-4, phiP333-4, phiCM117-3, phiG5-5, phiH14-2, phi23237-1, phi22825-1, phi22837-1, phi22838-1, phi22835-2, phi22841-1, phiG4-3, phiC58086, and phiSA14-639-2	*scn* gene
	phiAB333-4, phiP333-4, phiCM117-3, phiH14-2, phi22825-1, phi22837-1, phi22838-1, phi22835-2, phi22841-1, phiC58086, and phiSA14-639-2	*chp* gene
	phiCM117-3, and phiG5-5	*sep* gene
	phiG5-5	*sec* gene
	phiG4-3	*isdB* gene
	phiSA13-192-1	*xerD* gene
	phiG4-3 and phiH14-2	*yopX* gene
4	All prophages	*xerD* gene
5	All prophages	*sak* gene
	phiHD1410-3, phiH13-2, phiH4-3, phiH2-3, phiH1-3, phiH15-2, phiCM124-2, and phiCM101-2	*scn* gene
	phiH13-2, and phiH7-2	*sea* gene
	phiH4-3, phiH2-3, phiH1-3, phiH3-1, and phiH15-2	*chp gene*
	phiHD1410-3, phiH13-2, phiH4-3, phiH2-3, phiH1-3, phiH3-1, phiCM124-2, and phiCM101-2	*hlb* gene
	phiH15-2	*clpP ,* and *virE*
	phiH13-2	*yopX* gene
	phiH4-3	*eap*/*map* gene
6	All prophages	*clpP*, and *virE* genes
	phiRD.3-2	*geh* gene (lipase protein)
	phiH4-2, phiH2-2, phiH1-1, and phiI3-4	*xerD* gene
	phiH4-2, phiH2-2, and phiH1-1	*lukF-PV* and *lukS-PV* genes
	Phi1111205429-1, phiRD.60-3, phiFP_N239-1, and phiRD.3-2	*yopX* gene
7	phi909-4, and phi483-4	*lukE* (leukocidin related gene), enterotoxins encoding genes (*sei*, *sem*, *sen*, and *seo*)
	phi909-4	*seg*
	phi483-4	*seu*

The prophages belonging to clade 1, 6, and prophage phiH15-2 of clade 5 showed the presence of *clpP* and *virE* genes. Some of the prophages of clade 1 also showed the presence of PVL encoding genes (*lukF-PV* and *lukS-PV*), *yopX* gene belonging to Type-III secretion system, *sak* (staphylokinase) gene, *scn* (staphylococcal complement inhibitor) gene, and *chp* (chemotaxis inhibitory protein) gene. Besides, genes encoding a β-hemolysin/sphingomyelinase C (*hlb*), elastin binding protein (*ebp*), and tyrosine recombinase (*xerD* gene) were also detected in the prophages of Clade 1. The prophages phiCM124-1 of clade 2 showed the presence of iron-regulated surface determinants (*isd*) gene clusters (*isdA-isdH)*. The genes carried by the prophages of clade 3 mostly include *hlb*, *sak*, *scn,* and *chp*. In addition, *yopX* gene and genes that encode enterotoxins, *sep,* and *sec* were also detected in some prophages of clade 3. The *xerD* gene was also carried by the prophages phiH4-2, phiH2-2, phiH1-1, and phiI3-4 of clade 6, and clade 4. The prophages of clade 5 carried genes such as *sak, scn, chp,* and *hlb*. However, some prophages also showed the presence of a *sea* gene. *yopX* and *eap*/*map* genes. The prophages of clade 6 showed the presence of *lukF-PV* and *lukS-PV*, *clpP*, *xerD, virE,* and *yopX* genes*.* However, prophage phiRD.3-2 carried *geh* gene encoding lipase protein which might be a virulence factor and associated with the lysogenic conversion of *S. aureus*^30^. Most of the prophages especially phi909-4 and phi483-4 of clade 7 carried the toxin encoding genes viz., leukocidin-related gene (*lukE*), and enterotoxin genes (*seg*, *sei*, *sem*, *sen*, and *seo*). These prophages have genes that encode for the HNH endonuclease, a key component of phage DNA packaging machines^1,31,32^, and VRR-NUC domain protein (virus-type replication-repair nuclease). The variation in virulence factor encoding genes in prophages within each clade and among clades showed the genomic diversity of prophages, evolution, and the emergence of highly pathogenic *S. aureus* strains.

## Discussion

*S. aureus* causes moderate to severe infections in humans and animals^5,6^. The *S. aureus* strains that infect animals can be a potential risk of human zoonoses and a threat to public health^9,10,33^. The transmission of *S. aureus* strains from animals to humans occurs commonly^34^. Animals-associated *S. aureus* strains may spread to the human population through various routes such as contact with contaminated meat products, or infected farmers, butchers, and veterinary staff. Also, the contaminated effluent release from the animal farmhouses or veterinary hospitals could be another route for the transmission of *S. aureus* from animals to humans^35^. The *S. aureus* with CC398 (ST398) and CC151 (ST151) are the most identified clone types of bovines in the European regions^6,36^. It was reported that these CCs are transmitted to humans and are considered to be an emerging zoonotic agent^37^. In addition, the excessive or improper use of antibiotics in veterinary hospitals and animal husbandry promote antibiotic-induced SOS response in *S. aureus strains*^38,39^. This response triggers the phage induction and escalates the frequency of phage-mediated horizontal gene transfer (HGT) between the animals and humans-associated *S. aureus* strains^40^. For this reason, we selected prophages of CC398 and CC151 strains associated with animals to compare with prophages of CC398 or other CCs strains associated with human infections.

The pathogenicity of *S. aureus* is mainly driven by the acquisition of MGE such as chromosome cassettes, insertional sequence element (IS), plasmids, genomic (νSa) or pathogenicity islands (SaPIs), prophages, integrative conjugative elements (ICEs), and transposons as these elements carry genes that encode proteins involved in antibiotic resistance, and pathogenicity^7,19,33,41^. Acquisition of prophages may lead to an increase of the genome plasticity and maintains the architecture of the *S. aureus* genome and facilitating the adaptation in diverse conditions during infection^42,43^. Besides, they confer novel virulence properties that lead to the expansion of the pathogenic spectrum and enhance the severities of human and animal *S. aureus* infections^13^.

In this study, we characterized and compared the intact prophage regions of *S. aureus* strains associated with various human and animal site infections of different geographical locations to understand their relatedness, genomic architectures, and pathogenicity of the host *S. aureus* strains. The analyzed prophage genomes are mosaic in their architecture with extensive variation in genome size, and GC content (Table S2). All the identified complete prophages were belonged to *Siphoviridae* family and having temperate lifestyles. Also, based on the genomic architecture, most of the analyzed prophages consist of five functional modules, which are also found in the *Siphoviridae* family (Figs. 3, 4)^2^. The gene contents varied between 29 and 132 CDS. These prophages were identified as dsDNA temperate phages and can integrate into the host’s chromosome during the infection and behave as “quiescent” prophages^44^. It was reported that large phage genomes have more gene insertions such as transposons, self-splicing introns, and homing endonucleases, and many genes with unknown functions between various structural protein genes^45^. Prophages often encode ‘morons’ that are not directly engaged in their replication but may be components of bacterial host conferring a benefit to their bacterial host^46^. It was also reported that temperate phages can recombine with other prophages in the host genome results in high variation in genes and genomic sizes in the *Siphoviridae* family^47^. All the prophage genomes carried by human and animal-associated *S. aureus* showed the mosaic-like structure indicating that the frequency of HGT among the prophages was high and facilitated the development of new variant phages which in-turn may lead to the emergence of new pathogenic *S. aureus* strains.

The phylogenetic analysis of identified prophages showed that the prophages of the same host were dispersed in different clades rather than appearing in a single clade (Fig. 1). This finding suggested each *S. aureus* strain carried two or more different prophages with unique features. It was reported that a bacterial cell owning one or more prophages is considered as a lysogen that provides immunity toward the infection by the same group of phages^48^.

Prophages are a part of the accessory genome in a bacterial genome; however, identified prophages themselves have a pan-genome of 107,158 bp size. Notably, identified prophages did not have a core-genome that is conserved among all prophages across their phylogeny (Fig. 2); such a similar finding was reported previously^49^. The presence of functional modules with low sequence similarity may be due to recombination of two or more prophages within host genomes or horizontal exchange of functional modules between related phages^50^. Furthermore, the presence of variable MGEs, bacterial genes, and unspecified genes in the genome of prophages which were thought to be acquired from the different *S. aureus* strains suggested that such prophages had undergone several HGT events which result in prophage genomes with high variation^45^, and rapid emergence of new phages^23,51^. The low sequence similarity in the identified prophage genomes made it difficult to generate their core-genome. To overcome this limitation, we performed clade/subclade-wise prophage genomes analyses based on gene-by-gene alignment at a finer synteny level. The prophages carried by *S. aureus* associated with human or bovine infections have relatively high genome sizes in comparison with prophages of *S. aureus* associated with dogs. In Fig. 3A synteny, phiAB333-3, and phiP333-3 showed the highest sequence identities, and their host *S. aureus* strains have the same *SCCme*c IVc type and ST8 and same geographical origin (France), but these prophages were carried by *S. aureus* associated with human skin and nares infections. Similarly, in Fig. 3B synteny, phiVET1913R-2, and phiVET1914R-1 showed the closest relationship in this clade, and these two prophages were from the same country origin (Netherlands) and their host *S. aureus* strains carried the same *SCCmec* Vc type and ST398, however, they were found in the *S. aureus* associated with throat and nasal infections. In clade 4, phiI3-1, and phiHD1410-1 found 100% sequence similarities, these prophages are carried by *S. aureus* of different geographical locations (Germany, and Denmark). However, their host strains were identified as MSSA and have the same CC30. Similarly, Fig. 3D synteny revealed the highest degree of sequence similarity between the prophages carried by *S. aureus* strains (*SCCmec* IVa /ST398) associated with bovine infections and prophage (phi23237-1) carried by *S. aureus* strain (*SCCmec* IVa /ST398) associated with dog infection. Remarkably, this showed the evidence of phage-mediated horizontal gene transfer between the *S. aureus* associated with bovine infections and the *S. aureus* associated with dog infection (subclade 3A; Fig. 3D). In the Fig. 4B synteny, the host strains of prophages (phi53180-1-4, and phi81629-1) were associated with bovine, and human infections, and occupied distant geographical locations (Italy, and Denmark), however, the host strains of these prophages carried the same *SCCmec* IVa type, and ST398 (CC398), as a result, their prophages revealed high sequence similarities. In clade 7B, the *S. aureus* strains isolated in Netherlands that were associated with bovine milk were identified as MSSA strains and both have the same ST151, as a result, their prophages showed high sequence similarities (Fig. 4D).

The phylogenetic analyses result showed no difference in clustering patterns of prophages carried by HA-MRSA and CA-MRSA strains. The prophages (phiG5-2, phiG5-3, phiG5-5) carried by HA-MRSA strain were clustered with other prophages of CA-MRSA strains in clade 1, subclade 3B, and subclade 7B. Besides, the prophages carried by MSSA strains showed high proximity among them and found clustered in subclade 3B (phiI3-2, phiC5086-2, phiSA13-192-2, and phiSA14-639-2), clade 4 (phiI3-1, and phiHD1410-1), clade 7 (phi483-3, phi909-4, phiC3489-2, and phiC5086-2) (Fig. 1). This similar clustering pattern of prophages is correlated with similar sequence types or clonal complexes of MSSA strains. Also, the prophages similarities between the MRSA strains are favored by the same *SCCmec* types, sequence types, or clonal complexes of their host strains. This study further suggested that the frequency of phage-mediated horizontal gene transfer is higher between the *S. aureus* strains with the same *SCCmec* type, sequence type, or clonal complex.

The highest prevalence of immune evasion encoding genes was observed in the prophages of clades 3 and 5, however, such genes were not present in the genomes of prophages of clades 6 and 7 (Fig. 5). The 46 prophages harbored by human-associated *S. aureus* showed the presence of 64 VFGs whereas the 19 prophages carried by animal-associated *S. aureus* showed the presence of 36 VFGs related to toxin production, immune evasion, adhesion, and enzyme production. The prophages carried by animal-associated *S. aureus* have 50.34% more VFGs than the prophages harbored by human-associated *S. aureus*. This observation suggests that the prophages carried by animal-associated *S. aureus* may have more pathogenic potential. The PVL toxin was present in 7 prophages carried by human-associated *S. aureus*. Leucotoxin (*lukE*) was found in 2 prophages harbored by animal-associated *S. aureus.* These genes were located between the phage lysin and the *attR* site and the phages showed an elongated head instead of icosahedral^23^. It has been reported that helper phage φSLT mediates the transfer of these genes from a PVL-positive to a PVL-negative *S. aureus* strain during positive lysogenic conversion^2^. This toxin has been implicated in the pathogenesis of severe necrotic infections of higher vertebrates^52^. In the present study, IEC (*sak*, *chp*, and *scn*) was identified in prophages harbored by both human and animal-associated *S. aureus* which are known for positive lysogenic conversion. This IEC can be easily transferred from one *S. aureus* strain to another by a diverse group of *hlb*-converting bacteriophages and contributes to the pathogenomic diversity and human niche-specific adaptation of *S. aureus* strains^53^. The IEC is highly human-specific, and it is assumed that the IEC-containing phages are less prevalent in animal isolates^19,54,55^ and are lost when *S. aureus* shifts its host from human to animal^13^. The disruption of chromosomal factors through phage integration was known as a lysogenic negative conversion and results in the inactivation of *geh*, and *hlb* genes^29^. The prophage, phiRd.3-2 of *S. aureus* associated with bovine infection possesses *geh* and it was reported that the integration of the *geh* gene causes negative lysogenic conversion in Staphylococcal phage L54a^56^. Our study observed the high prevalence of enterotoxin-gene cluster (*egc*) such as *seg, sei, sem, sen, seo,* and *seu* in the prophages of animal-associated *S. aureus.* This *egc* was identified mainly in phi483-3, and phi909-4 of clade 7 harboring the novel SaPIs (Fig. 5). It was reported that *egc* acts as a colonization factor thereby magnifying the commensal fitness and shows aggravating effects in bacteremia^12^. Besides, enterotoxin-encoding genes (*sea* and *sep*) were found in the prophages carried by human-associated *S. aureus* (clade 5 of Fig. 5) which are responsible for causing infections such as food poisoning, toxic shock syndrome, necrotizing fasciitis bullous impetigo, and chronic bovine mastitis^57^. The prophage phiCM124-1 showed the presence of *isd* gene clusters which are required by the *S. aureus* for iron acquisition and resulting in lysis of erythrocytes in humans and animals^58^. Also, extracellular adherence protein encoded by *eap* gene observed in phiH14-1, phiH3-2, is reported to facilitate adherence of *S aureus* to host extracellular matrix components and prevents inflammation, wound healing, and angiogenesis^59^. Besides, the presence and distribution of different virulence factors encoding genes among the prophages of various clades suggested that either the presence of these factors is dependent on the host environmental condition or these factors allow the host bacteria to adapt to different environmental niches^60^. The prophages carried by human-associated *S. aureus* strains of different serotypes obtained from different geographical locations scattered themselves in all the clades, suggesting that these phages have a wide distribution across the European regions. A similar finding was also reported earlier in prophages of *Streptococcus suis*^60^. The prophages carried by animal-associated *S. aureus* also showed their presence in different clades viz., clade 3A, clade 6, and clade 7B (Fig. 1) and displayed slightly different clustering patterns compared to the clustering pattern of human-associated *S. aureus* prophages. Overall, this study demonstrates that the presence of prophages in the genome of *S. aureus* associated with both humans and animals causes genetic variations in the bacterium, confers antibiotic resistance, and helps the bacterium to adapt to hostile conditions and that in turn increases its pathogenicity.

The present study was performed to characterize and compare the prophages carried by humans and animal-associated *S. aureus* strains reported from different geographical locations as well as different infection sites. This comparative study revealed the diversity of prophages of *S. aureus* associated with humans or animals. In our study, all the CC398 strains were identified in MRSA strains and showed high prevalence in animal-associated *S. aureus* strains. The prophages carried by CC398 clone of animals and humans associated with *S. aureus* strains showed disperse in different clades (Fig. 1). The presence of similar genetic elements in the prophages isolated from *S. aureus* associated with animals and humans suggested that prophages may have played a major role in the epidemiological changes. The appearance of the mosaic nature of prophage genomes suggested the occurrence of genetic exchange among the *S. aureus* strains via phages. Also, the presence of VFGs in the genomes of prophages supports *S. aureus* to adapt in different environmental niches, promote the pathogenesis and facilitate their evolution. The IEC was identified in both prophages harbored by human and animal-associated *S. aureus* which are a human niche-specific adaptation of *S. aureus* strains. The IEC is highly human-specific, however, our finding revealed that the presence of IEC could not differentiate between phages of human and animal-associated *S. aureus.* The presence of various virulence factors in the genomes of prophages of animal-associated *S. aureus* suggested that these prophages could have more pathogenic than the prophages of human-associated *S. aureus.*

This study also showed that the prophages carried by human-associated *S. aureus* strains with different serotypes and from different geographical locations scattered in all the clades, indicating that these phages have a wide distribution across the European regions. Comparative studies of prophages carried by human and animal-associated *S. aureus strains* have very crucial importance for the investigation of *S. aureus* transmission from human to animal and *vice-versa*, as well as to gain a better understanding of their evolutionary relationships, and diversity.

## Methods

### Data collection and Identification of prophages

A total of 60 whole genomes of *S. aureus* strains reported to cause human and animal infections across the European regions were used in this study. Of these 60 whole genome sequences of *S. aureus* strains, 54 were retrieved from the NCBI database and additional six genome assemblies of *S. aureus* were from our previous studies^25,27^. The *S. aureus* strains used in this study originated from Austria (n = 7), Denmark (n = 5), France (n = 12), Germany (n = 11), Hungary (n = 3), Italy (n = 9), Netherlands (n = 11), and Spain (n = 2). The genome sequences were analyzed for *SCCmec* types^61^, and Multilocus sequence Type (MLST)^62^ using a web-based server provided by the Center for Genomic Epidemiology.

The prophage sequences or phages associated with these genomes were analyzed for their diversity based on the geographic location and nature of *S. aureus* infected hosts. The details of the whole genomes used in this study are presented in Table S1.

### General genomic features of the putative prophages

PHAge Search Tool Enhanced Release (PHASTER) algorithm was used to identify and annotated prophage sequences from 60 *S. aureus* genomes^63^. Prophage sequences with PHASTER score ≤ 70 is considered as incomplete, score between 70 and 90 is regarded as questionable, while the score ≥ 90 is considered as intact/complete prophages. The intact prophage genome sequences were extracted from their respective host *S. aureus* genomes to predict open reading frames (ORFs) using GeneMarkS^64^ and the predicted genes were analyzed against the NCBI database using BLASTP^65^. The identified intact prophages were classified for their lifestyles using PHACTS (Phage Classification Tool Set)^66^. The tRNAscan-SE v.1.21 was used to decipher the tRNA-coding regions in the prophage sequences^67^. Further, the intact/complete prophage genomes were re-annotated using prokka^68^ 1.14.

### Sequence clustering and phylogenetic relationship of the prophages

A total of 65 intact prophage sequences of *S. aureus* strains were identified by PHASTER. The 65 intact prophage nucleotide sequences were subjected to Multiple sequence Alignment using Fast Fourier Transform (MAFFT) version^69^ v7.475. Further, the aligned sequences of intact prophage nucleotide sequences were run on SplitsTree4 software^70^ to generate the hierarchical clusters and displayed as a phenogram using the BioNJ algorithm^71^.

### Comparative genomic analyses of S. aureus prophages

The identified intact prophage sequences were in silico analyzed for the identification of ARGs and VFGs using CARD^72^ and VirulenceFinder-2.0 Server^73^, respectively. The heatmap was generated to illustrate the presence or absence of VFGs using Morpheus^74^. The intact prophage sequences were used for a pan-genome comparison using the TBLASTX and prophage phiH14-1 as a seed genome in Gview server^75^ (https://server.gview.ca/). Furthermore, the prophage sequences belonging to each cluster or clade were analyzed for core and accessory genomes using Spine and AGEnt version 0.3.1 webserver^76^. The number of core and accessory genomes of prophages in the gene pool of each cluster or clade was extracted, and a flowerplot was generated using plotrix in RStudio 1.3 (RStudio_Team, 2020)^77^. The intact prophage sequences that comprised each cluster were aligned using Easyfig version^78^ 2.2.3. Easyfig alignments were performed on selected groups of prophages based on their clusters to show regions of sequence identity and their closest phages (phiNM-3, phiStauST398-3, and phi2958PVL) defined by PHASTER.

### Use of human subjects or animals in research

This article does not contain any studies involving human and animal participants performed by any of the authors.

## Supplementary Information

Supplementary Information 1.

Supplementary Information 2.

## Data Availability

The following information was supplied regarding data availability: The *S. aureus* genome sequences used in this study are available at https://www.ncbi.nlm.nih.gov/nucleotide/ or https://www.patricbrc.org/remote under the accession number given in Table S1.
