# Supplementary material for: Comparative analysis of prophages carried by human and animal-associated Staphylococcus aureus strains spreading across the European regions
Source: Sci Rep. 2021 Sep 23;11:18994. doi: 10.1038/s41598-021-98432-8 (PMC8460829; doi:10.1038/s41598-021-98432-8)
Supplement: Supplementary file 1 — Supplementary Information 1. [file 41598_2021_98432_MOESM1_ESM.pdf]

Comparative analysis of prophages carried by human and animal-associated *Staphylococcus aureus* strains spreading across the European regions.

Romen Singh Naorem [1], Gunajit Goswami[2], Schneider Gyorgy[3], and Csaba Fekete [1*]

[1]Department of General and Environmental Microbiology, Institute of Biology and Sport Biology, University of Pécs, Pécs, Hungary.

[2]Multidisciplinary Research Unit, Jorhat Medical College and Hospital, Jorhat, Assam, India

[3]Department of Medical Microbiology and Immunology, Medical School, University of Pécs, Pécs, Hungary.

Running title: Comparison of *S. aureus* prophages

Keywords: *Staphylococcus aureus*, Prophage, Comparative genomics, Pan-genome analysis, Phylogenetic tree analysis.

***Correspondence:** Csaba Fekete, Department of General and Environmental Microbiology, University of Pecs, Ifjusag utja. 6, Pécs 7624, Hungary.

Phone: +36-72503600

Email: fekete@gamma.ttk.pte.hu

ORCID: 0000-0001-9430-4643

ORCID: 0000-0002-6562-6106

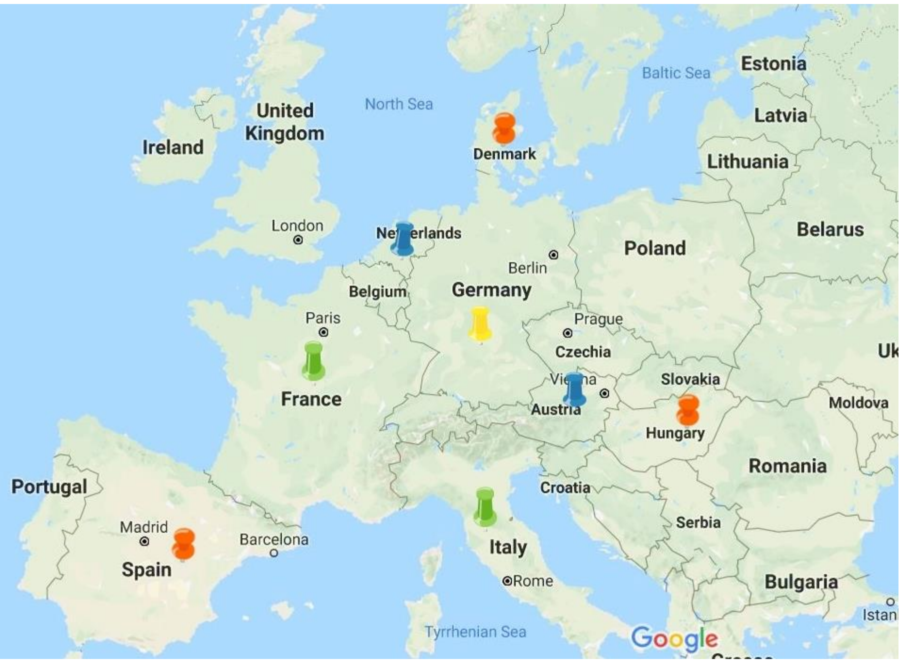

Supplementary Fig. S1 Map showing the geographical locations of selected *S. aureus* strains. The geographical locations of *S. aureus* strains used in this study were pin with blue, green, orange, and yellow colors on the map. The map was retrieved from Google Maps (Google Maps Available at: https://www.google.co.in/maps/place/Europe/@47.103851,17.4032248,4.59z/data=!4m5!3m4!1s0x46ed8886cfadda85:0x72ef99e6b3fcf079!8m2!3d54.5259614!4d15.2551187. (Accessed: 26th November 2019)) and locations were marked using PhotoScape V3.7 (http://www.photoscape.org/).

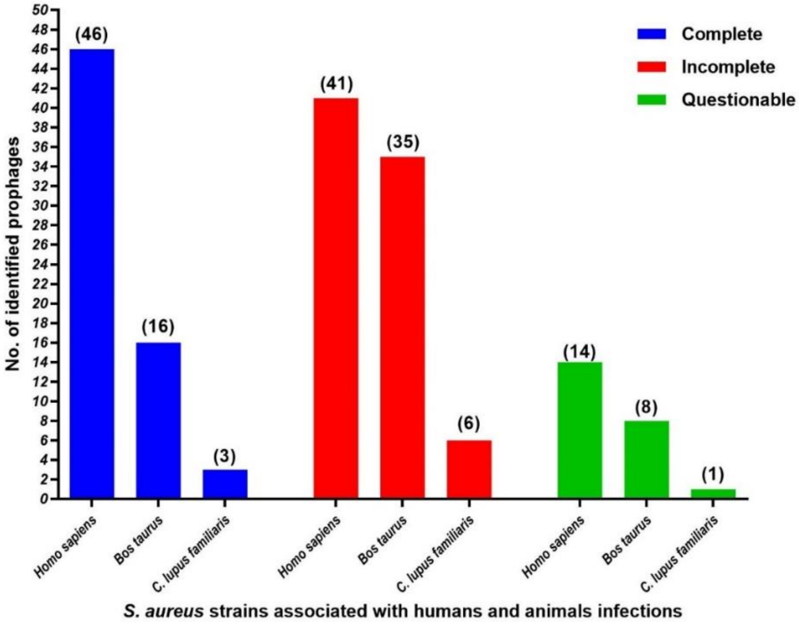

Supplementary Fig. S2 Prophages distribution in *S. aureus* genomes. The graph was generated using GraphPad Prism 6 (Graphpad Software INC, CA, USA).

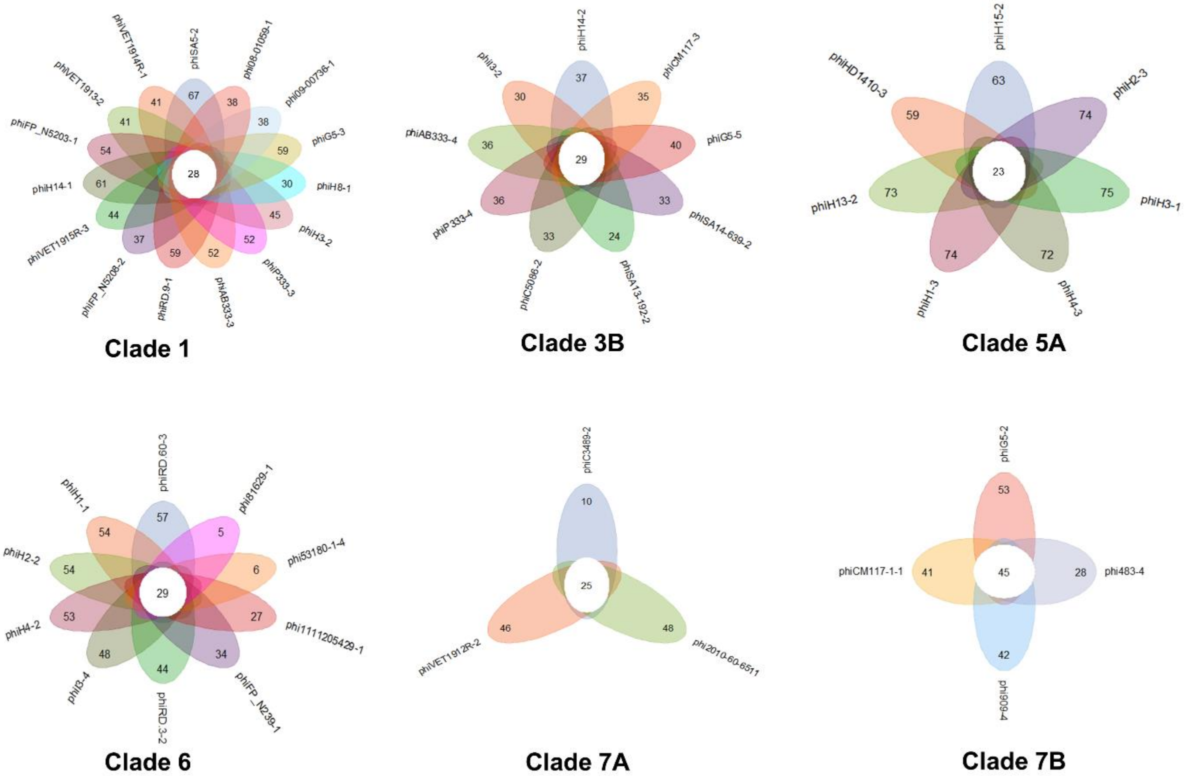

Supplementary Fig S3. **Flower-plot showing the genes of core-genome (center) and accessory genes (petal). Each prophage is represented by an oval with a different color.** In clade 1, the pan-genome has 168 CDS, and the core-genome has 28 CDS **(Clade 1).** In subclade 3B, the pan-genome has 111 CDS, and the core-genome has 29 CDS **(Clade 3B)**. In subclade 5A, the pan-genome has 159 CDS, and the core-genome has 23 **(Clade 5A)**. Clade 6 has a pan-genome comprised of 170 CDS, and the core-genome has 29 CDS **(Clade 6)**. Subclade 7A formed pan-genome consists of 86 CDS, and core-genome size has 25 CDS **(Clade 7A)**. In subclade 7B, the pan-genome has 115 CDS, and the core-genome consists of 45 **(Clade 7).**

Table S2. General features of prophages extracted from the *S. aureus* strains

Sl. No.	Prophages	Size (Kb)	GC%	CDS	Located region	Score	Host	*SCCmec Type*	*S. aureus* Type	MLST Type	Clonal Complex (CC)	GenBank Accession	Origin
1	phi08-01059-1	46.9	33.23	65	11485-58400	Intact (130)	*S. aureus 08-01059*	Vc	CA-MRSA	ST398	CC398	JJEX00000000	Human
2	phi09-00736-1	46.9	33.23	66	11416-58331	Intact (130)	*S. aureus 09-00736*	Vc	CA-MRSA	ST398	CC398	JJEM00000000	Human
3	phiHD1410-1	33.8	32.5	32	287318-321167	Intact (95)	*S. aureus strain HD1410*	Absent	MSSA	ST34	CC30	NXFH01000000	Human
4	phiHD1410-2	59.7	33.5	65	17033-76828	Intact (120)							
5	phiHD1410-3	44.9	32.9	64	56719-101698	Intact (100)							
6	phi1111205429-1	41.2	33.83	56	3-41249	Intact (130)	*S. aureus 1111205429*	Vc	CA-MRSA	ST398	CC398	JJDO00000000	Human
7	phiVET1915R-3	48	33.69	73	243-48313	Intact (140)	*S. aureus VET1915R*	Vc	CA-MRSA	ST398	CC398	JIFU00000000	Human
8	phiVET1914R-1	47.3	33.67	67	137706-185045	Intact (130)	*S. aureus VET1914R*	Vc	CA-MRSA	ST398	CC398	JIFV00000000	Human
9	phiVET1913R-2	47.3	33.67	66	239198-286537	Intact (130)	*S. aureus strain VET1913R*	Vc	CA-MRSA	ST398	CC398	JIFW00000000	Human
10	phiVET1912R-2	43.2	34.86	72	30927-74209	Intact (120)	*S. aureus strain VET1912R*	IVa	CA-MRSA	ST398	CC398	JIFX00000000	Human
11	phi81629-1	30.4	33.78	35	3-30465	Intact (100)	*S. aureus 81629*	IVa	CA-MRSA	ST398	CC398	JJAH00000000	Human
12	phiI3-1	33.8	32.5	31	302540-336406	Intact (95)	*S. aureus strain I3*	Absent	MSSA	ST30	CC30	MVFW00000000	Human
13	phiI3-2	41.7	33.4	60	1-41759	Intact (108)							
14	phiI3-4	63.3	32.8	65	33452-96810	Intact (110)							
15	phiCM101-2	56.7	32.2	68	15026-71738	Intact (130)	*S. aureus strain CM101*	IVc	CA-MRSA	ST101	CC101	PZVM01000000	Human
16	phiCM124-1	64.8	33.8	64	21328-86146	Intact (150)	*S. aureus strain CM124*	IVa	CA-MRSA	ST1	CC1	PZUR01000000	Human
17	phiCM124-2	60.2	32.5	78	32094-92386	Intact (140)							

18	phiCM117-1	49.5	34.2	63	153991-203576	Intact (140)	*S. aureus strain CM117*	IVc	CA-MRSA	ST5	CC5	PZUX01000000	Human
19	phiCM117-3	46.3	32.8	65	10397-56750	Intact (100)							
20	phiCM112-3	28.1	34.4	33	729-28851	Intact (110)	*S. aureus strain CM112*	IVc	CA-MRSA	ST5	CC5	PZVC01000000	Human
21	phiCM60-3	28.6	35.1	37	257-28905	Intact (100)	*S. aureus strain CM60*	Vc	CA-MRSA	ST239	CC8	PZWT01000000	Human
22	phiH4-2	62.2	32.6	73	187368-249650	Intact (140)	*S. aureus strain H4*	IVa	CA-MRSA	ST8	CC8	NKCT00000000	Human
23	phiH4-3	56.6	32	67	3980-60610	Intact (100)							
24	phiH2-2	63.2	32.5	73	187298-250551	Intact (140)	*S. aureus strain H2*	IVa	CA-MRSA	ST9	CC9	NKCV00000000	Human
25	phiH2-3	59.9	32.2	74	31830-91747	Intact (110)							
26	phiH8-1	44.2	33.2	55	17487-61726	Intact (120)	*S. aureus strain H8*	IVa	CA-MRSA	ST11	CC5	NKCP00000000	Human
27	phiH3-1	59.9	32.4	70	1136-61074	Intact (110)	*S. aureus strain H3*	IVa	CA-MRSA	ST8	CC8	NKCU00000000	Human
28	phiH3-2	53.5	33	63	3382-56909	Intact (120)							
29	phiH1-1	63.2	32.5	73	187368-250621	Intact (140)	*S. aureus strain H1*	IVa	CA-MRSA	ST10	CC10	NKCW00000000	Human
30	phiH1-3	59.9	32.2	74	31829-91746	Intact (110)							
31	phiH7-2	24.8	34.4	29	3-24873	Intact (100)	*S. aureus strain H7*	IVa	CA-MRSA	ST8	CC8	NKCQ00000000	Human
32	phiAB333-3	60.4	32.6	73	1536165-1596570	Intact (140)	*S. aureus strain AB333*	IVc	CA-MRSA	ST8	CC8	LSGN01000000	Human
33	phiAB333-4	45.8	32.8	66	162378-208180	Intact (100)							
34	phiP333-3	60.4	32.6	73	1534491-1594896	Intact (140)	*S. aureus strain P333*	IVc	CA-MRSA	ST8	CC8	LSGO01000000	Human
35	phiP333-4	45.8	32.8	66	88453-134255	Intact (100)							
36	phiSA13-192-1	32.1	34	54	524-32652	Intact (110)	*S. aureus subsp. aureus strain SA13-*	Absent	MSSA	ST398	CC398	LNJF00000000	Human

37	phiSA13-192-2	33.8	33.5	53	515-34393	Intact (110)	*192*						
38	phiSA14-639-2	44.5	33	65	13727-58297	Intact (120)	*S. aureus subsp. aureus strain SA14-639*	Absent	MSSA	ST398	CC398	LNJO00000000	Human
39	phiG4-3	87.8	33.92	132	1944955-2032780	Intact (150)	*S. aureus subsp. aureus strain SA G6*	I	HA-MRSA	ST228	CC5	RAHA00000000	Human
40	phiG5-2	61.2	33.59	90	682931-744225	Intact (150)	*S. aureus subsp. aureus strain SA G8*	II	HA-MRSA	ST225	CC5	QZFC00000000	Human
41	phiG5-3	68.2	32.59	79	1517828-1586108	Intact (130)							
42	phiG5-5	51.8	32.22	59	2040204-2092008	Intact (106)							
43	phiH13-2	56.4	32.16	66	1013609-1070087	Intact (100)	*S. aureus subsp. aureus strain SA H27*	IVa	CA-MRSA	ST22	CC22	CP032468	Human
44	phiH14-1	73.3	32.75	73	1502314-1575621	Intact (120)	*S. aureus subsp. aureus strain SA H29*	IVa	CA-MRSA	ST8	CC8	CP032161	Human
45	phiH14-2	45.5	32.94	64	2018439-2063982	Intact (100)							
46	phiH15-2	58.7	33.56	70	736189-794911	Intact (150)	*S. aureus subsp. aureus strain SA H32*	IVa	CA-MRSA	ST22	CC22	RAHP00000000	Human
47	phi2010-60-6511	46.7	34.76	68	222914-269656	Intact (120)	*S. aureus 2010-60-6511-10*	IVa	LA-MRSA	ST398	CC398	JJCG00000000	Animal
48	phi22835-2	44.7	33	68	98989-143737	Intact (100)	*S. aureus 22835*	IVa	LA-MRSA	ST398	CC398	JJBV00000000	Animal
49	phi53180-1-4	31.5	33.72	36	82-31604	Intact (110)	*S. aureus 53180-1*	IVa	LA-MRSA	ST398	CC398	JJAW00000000	Animal
50	phiFP_N239-1	46.4	33.55	64	142176-188632	Intact (110)	*S. aureus FP_N239*	IVa	LA-MRSA	ST398	CC398	JIYQ00000000	Animal
51	phiFP_N5203 OX-1	63.7	33.15	71	1137-64873	Intact (140)	*S. aureus FP_N5203 OX*	V	LA-MRSA	ST398	CC398	JIYP00000000	Animal
52	phiFP_N5208 OX-2	48.1	33.49	62	239490-287619	Intact (120)	*S. aureus FP_N5208 OX-2*	IV	LA-MRSA	ST398	CC398	JIYO00000000	Animal
53	phi483-4	55.1	33	80	35402-90527	Intact (150)	*S. aureus 483*	Absent	MSSA	ST151	CC151	QFCY01000000	Animal
54	phi909-4	55	33.1	80	35713-	Intact	*S. aureus 909*	Absent	MSSA	ST151	CC151	QFCZ01000000	Animal

					90759	(140)							
55	phi22825-1	44.7	33	68	63597-108345	Intact (100)	*S. aureus 22825*	IVa	LA-MRSA	ST398	CC398	JJBW00000000	Animal
56	phi22837-1	44.7	33	68	91414-136162	Intact (100)	*S. aureus 22837*	IVa	LA-MRSA	ST398	CC398	JJBU00000000	Animal
57	phi22838-1	44.7	33	68	63604-108352	Intact (100)	*S. aureus 22838*	IVa	LA-MRSA	ST398	CC398	JJBT00000000	Animal
58	phi22841-1	44.7	33	66	63656-108404	Intact (100)	*S. aureus 22841*	IVa	LA-MRSA	ST398	CC398	JJBS00000000	Animal
59	phiSa52-2	73.2	33	74	40846-114049	Intact (140)	*S. aureus Sa52*	IVa	LA-MRSA	ST398	CC398	NBNF00000000	Animal
60	phiRd.3-2	57.2	33.99	59	26855-84075	Intact (110)	*S. aureus Rd.3*	IVa	LA-MRSA	ST398	CC398	JIWZ00000000	Animal
61	phiRd.9-1	70.2	33.2	69	4314-74563	Intact (140)	*S. aureus Rd.9*	Vc	LA-MRSA	ST398	CC398	JIWT00000000	Animal
62	phiRd.60-3	67.6	33.6	63	7053-74719	Intact (130)	*S. aureus Rd.60*	Vc	LA-MRSA	ST398	CC398	JIWV00000000	Animal
63	phi23237-1	44.7	33	68	63656-108404	Intact (100)	*S. aureus 23237*	IVa	LA-MRSA	ST398	CC398	JJBO00000000	Animal
64	phiC3489-2	29.2	35.38	38	2-29221	Intact (140)	*S. aureus C3489*	Absent	MSSA	ST398	CC398	JIZE00000000	Animal
65	phiC5086-2	44.5	33.11	65	320203-364773	Intact (120)	*S. aureus C5086*	Absent	MSSA	ST398	CC398	JIYX00000000	Animal
